# Preoperative Diagnostic Strategy for Parotid Gland Tumors Using Diffusion-Weighted MRI and Technetium-99m Pertechnetate Scintigraphy: A Prospective Study

**DOI:** 10.1371/journal.pone.0148973

**Published:** 2016-02-05

**Authors:** Masahiro Kikuchi, Sho Koyasu, Shogo Shinohara, Yukihiro Imai, Megumu Hino, Yasushi Naito

**Affiliations:** 1 Department of Otolaryngology-Head and Neck Surgery, Kobe City Medical Center General Hospital, Kobe, Hyogo, Japan; 2 Department of Radiology, Kobe City Medical Center General Hospital, Kobe, Hyogo, Japan; 3 Department of Clinical Pathology, Kobe City Medical Center General Hospital, Kobe, Hyogo, Japan; School of Medicine, Fu Jen Catholic University, TAIWAN

## Abstract

**Objective:**

Fine needle aspiration cytology (FNAC) for diagnosis of a parotid gland tumor is widely used but its sensitivity is low and non-diagnostic rate is relatively high. In contrast, core needle biopsy (CNB) has a higher sensitivity and lower rate of sampling errors but has a higher risk of injury to adjacent organs such as facial nerve than FNAC. Screening of patients with parotid gland tumors to identify cases of pleomorphic adenoma (PA) and Warthin tumor (WT) may allow CNB to be confined to patients without PA and WT. We established an algorithm for preoperative diagnosis and management of parotid gland tumor using diffusion-weighted MRI and ^99m^Tc pertechnetate scintigraphy. This algorithm was developed with the goal of maximal reduction of the number of patients in whom CNB is required. The purpose of the study is to validate our algorithm prospectively.

**Methods:**

A prospective study was conducted in 71 cases who were newly diagnosed with parotid gland tumor and 53 cases were enrolled in the study. In the algorithm, PA (high apparent diffusion coefficient (ADC) _mean_≥1.5×10^−3^ mm^2^/s) and non-PA (low ADC_mean_<1.5×10^−3^ mm^2^/s) cases are first distinguished based on the ADC_mean_ on diffusion-weighed MRI. Second, among suspected non-PA cases, WT and non-WT are distinguished using technetium-99m pertechnetate scintigraphy. CNB is then performed only in probable non-PA and non-WT cases.

**Results:**

Although CNB was only required in 40% (21/53) of all cases, we made a preoperative histopathological diagnosis with an accuracy of 87% (46/53) and we correctly diagnosed whether a tumor was benign or malignant with an accuracy of 96% (51/53). Preoperative surgical planning had to be changed during surgery in only one case (2%)

**Conclusions:**

Our algorithm is valuable in terms of clinical practice with highly potential for preoperative diagnosis and with less risk of CNB procedure.

## Introduction

Surgical excision of a parotid gland tumor has a risk of facial nerve injury. Thus, an accurate preoperative histopathological diagnosis of a parotid lesion is important in establishing a surgical indication and for preoperative surgical planning. Fine needle aspiration cytology (FNAC) is widely used for diagnosis of a parotid gland lesion and can predict if the lesion is benign or malignant with an accuracy of 81–98%[1]. The advantages of this method are well documented[1, 2], but its limitations include low sensitivity (about 80%[3]), compared with its high specificity (about 95%[3]), and a relatively high non-diagnostic rate of about 10% due to sampling errors[3–5].

Core needle biopsy (CNB) has been introduced as an alternative to FNAC for preoperative assessment of a salivary gland lesion[3, 6, 7]. In a recent systematic review and meta-analysis, Witt et al. found that CNB of salivary gland lesions has a high sensitivity of 96% (95% confidence interval (CI) = 87–99%), a high specificity of 100% (95% CI = 84~100). The non-diagnostic rate for CNB is only 1.6%[3]. Therefore, CNB may be an alternative with a better diagnostic outcome compared to FNAC. In contrast, CNB may have the higher possibility of damaging adjacent organs such as the facial nerve or vessels and seeding tumor cells, because CNB needs a larger needle than FNAC. Although CNB has been reported to be safe with no notable complications, this may be due to the publication bias. In addition, this risk also increases for tumors located in a deep portion of the parotid gland[8]. To reduce this risk as much as possible, it would be advantageous to select patients in whom CNB is required[9], based on a high probability of a malignant tumor. Malignant tumors comprise only about 20% of all parotid gland tumors, and therefore effective pre-test screening for a malignant parotid gland tumor is required for selection of patients who should undergo CNB.

A low apparent diffusion coefficient (ADC) in diffusion-weighted MRI indicates decreased diffusion of water molecules in tissue. Therefore, a tumor with low ADC contains a greater number of cellularity than the one with high ADC_._ This means that ADCs of malignant tumors tend to be lower than those of benign tumors, and thus the ADC is useful to detect malignant head and neck tumors[10, 11]. We took advantage of these features of ADC quantification and hypothesized that selection of patients who need CNB for parotid gland tumors may be possible by evaluating the tumors using the ADC value derived from diffusion MRI.

Pleomorphic adenoma (PA) is the most frequent benign tumor of the parotid gland and usually has a myxoid matrix retaining an abundance of free water molecules, which leads to a high ADC[12–16]. This indicates that CNB may be spared in patients with tumors with a high ADC. Warthin tumor (WT), the second most frequent benign parotid gland tumor, has high cellularity due to epithelial proliferation with lymphocytic infiltration[17], which leads to a low ADC[13, 14, 17, 18]. This prevents retaining use of ADC for differentiation of WT from malignant tumors because the ADC of WT usually overlaps with that of a malignant tumor[13, 14, 17, 18]. However, technetium-99m (^99m^Tc) pertechnetate scintigraphy is useful for diagnosis of WT[19, 20]. Hence, we additionally hypothesized that the candidate for CNB can be further reduced by screening WT with ^99m^Tc pertechnetate scintigraphy among tumors with a low ADC value.

To verify these hypotheses, we established an algorithm for preoperative diagnosis and management of parotid gland tumor using diffusion-weighted MRI and ^99m^Tc pertechnetate scintigraphy. This algorithm was developed with the goal of minimizing the candidate for CNB. The purpose of the current study is to validate this algorithm prospectively.

## Materials and Methods

### Patients

A prospective study was conducted in 71 patients who were newly diagnosed with parotid gland tumor between June 2011 and September 2013 at our hospital. The inclusion criteria were a newly diagnosed tumor in the parotid gland detected on MRI with contrast enhancement. The exclusion criteria were (a) surgery not performed (n = 3; rejected by patient, n = 3; poor condition of patient, n = 2; f/u because of small tumor with the largest diameter <10 mm); (b) <20 years old (n = 1); (c) pure cyst or tumor with a necrotic/cystic component >50% of the total lesion volume (n = 4); (d) MRI or ^99m^Tc scintigraphy not performed (n = 2); (e) pain or facial paralysis that were strongly suggestive of malignancy (n = 1); and (f) withdrawal of consent (n = 2). Thus, a total of 53 patients were enrolled in the study.

Bulk necrosis and cystic degeneration of tumors usually increase diffusivity by the free movement of water molecules within those histological changes and may contribute to a higher tumor ADC than the actual ADC for characterization of tumors[16]. This is why tumor with bulk necrotic/cystic component was excluded in this study. The histopathology of the 4 excluded cystic tumors confirmed by surgery were WT (n = 1), PA (n = 1), lymphoepithelial cyst (n = 1) and salivary ductal cyst (n = 1).

Kobe City Medical Center General Hospital Institutional Review Board approved the study (the approval number is 1105–8) and written informed consent for participation was signed by all 53 patients.

### Algorithm for preoperative diagnosis and management of parotid gland tumor

**Fig 1:**
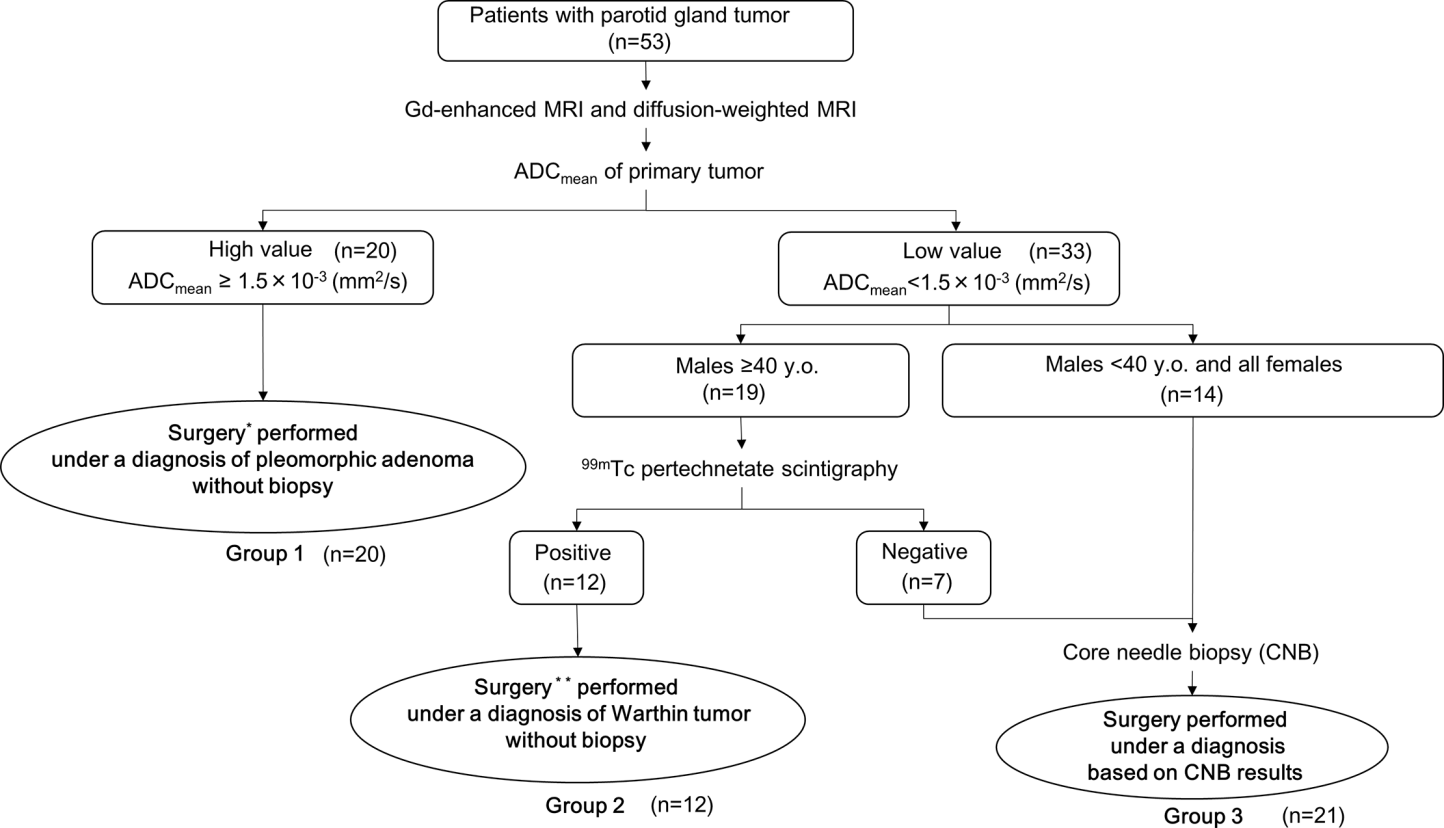
Algorithm for preoperative diagnosis and surgical planning for parotid gland tumors.

We formalized an algorithm (Fig 1) for preoperative diagnosis and surgical planning. First, an average ADC value (ADC_mean_) was calculated for each tumor.

In previous reports[12, 14], DWI is useful for distinguishing PA and non-PA cases with an ADC_mean_ cut-off of 1.315 to 1.4×10^−3^ mm^2^/s. Based on these cut-off values, we established our cut-off of 1.5×10^−3^ mm^2^/s. Patients with tumors with high ADC_mean_ (≥1.5×10^−3^ mm^2^/s) were categorized in group 1, based on strong suspicion of a benign tumor (PA), and underwent surgery (partial parotidectomy with exposure and preservation of the facial nerve) without preoperative biopsy. Patients with tumors with low ADC_mean_ (<1.5×10^−3^ mm^2^/s) underwent ^99m^Tc pertechnetate scintigraphy (males ≥40 years old) or CNB (males <40 years old and all females). Patients with a positive result in ^99m^Tc pertechnetate scintigraphy were categorized in group 2, based on diagnosis of probable WT, without preoperative biopsy and underwent surgery (enucleation or extracapsular dissection without exposing the facial nerve). Patients with a negative result in ^99m^Tc pertechnetate scintigraphy underwent CNB. All patients who received CNB were categorized in group 3 and underwent surgery based on the histopathological results of CNB.

### MRI analysis

Diagnostic MRI scans were performed using a 1.5 T scanner (Avanto, Siemens AG, Healthcare Sector, Erlangen, Germany). Non-contrast enhanced T1-weighted images (T1WI), T2-weighted images (T2WI), and diffusion-weighted images (DWIs) with b-values of 0 and 1000 s/mm^2^ were acquired in the axial plane. T2WI with the fat saturation technique using short inversion time inversion-recovery (STIR) were acquired in the coronal plane. Transaxial and coronal plane of T1WI with gadolinium contrast agent were also acquired. For each DWI sequence, a pixel-by-pixel ADC map was automatically calculated, with the ADC value expressed in mm^2^/s. A region of interest (ROI) drawn freehand including as large an area of tumor as possible was manually placed on each slice of the tumor with the largest diameter on an axial ADC map. Localization of each ROI was confirmed using morphologic T1WI, T2WI, and contrast-enhanced images with visual exclusion of large cystic or necrotic areas and large vessels such as the retromandibular vein. Delineation of tumors on ADC maps was performed by a board-certified radiologist (K.S.) with consensus with a board-certified head and neck surgeon (K.M.), and the ADC_mean_ per ROI was measured.

### ^99m^Tc pertechnetate scintigraphy

Twenty minutes after intravenous administration of 185 MBq ^99m^Tc pertechnetate, planar scintigraphy imaging (anteroposterior image and lateral images on both sides) of the parotid gland was performed. After the first scans, salivary gland secretion was stimulated with oral Cinal (1 g: ascorbic acid 200 mg, calcium pantothenate 3 mg). Two minutes after Cinal administration, second scans of washout planar images were acquired. Immediately after these scans, SPECT using a dual-head gamma camera (Millennium VG, GE Healthcare; until June 2011) or SPECT/CT using a hybrid SPECT/CT scanner combining a dual-head gamma camera and a helical CT scan for attenuation correction (Infinia Hawkeye 4, GE Healthcare; from July 2011) was performed. Images were prospectively evaluated based on consensus by board-certified nuclear medicine physicians and radiologists (H.M. and K.S.).

### Core needle biopsy

CNB was performed freehand (for superficial large tumors) or with ultrasound guidance (for deep or small tumors) by board-certified head-and-neck surgeons (K.M. and S.S.). An 18-gauge (1.2 mm) needle was used without local anesthetic. CNB was attempted once or twice. Neither a cytologist nor a technician was present in the outpatient clinic at the time of sampling. CNB was performed manually using a 20-ml sterile disposable plastic syringe with the 18-gauge needle held by an aspiration gun, rather than with a commercial needle device or automated biopsy device.

### Histopathological analysis

Specimens were stained with hematoxylin-eosin and evaluated by one experienced pathologists (I.Y.).

### Validation of the algorithm

The accuracy of preoperative histopathological diagnosis was evaluated as the primary endpoint. To determine the diagnostic accuracy for PA, sensitivity, specificity, positive predictive value (PPV), and negative predictive value (NPV) were calculated for classification of patients into group 1 (suspected PA). Similarly, the diagnostic accuracy for WT was evaluated based on the sensitivity, specificity, PPV, and NPV for classification of patients into group 2 (suspected WT). CNB was performed for patients in group 3. The accuracy for determining whether the tumor was benign or malignant and the diagnostic accuracy of histopathology were analyzed respectively.

## Results

### Patient characteristics, preoperative data, and postoperative histopathology

**Fig 2:**
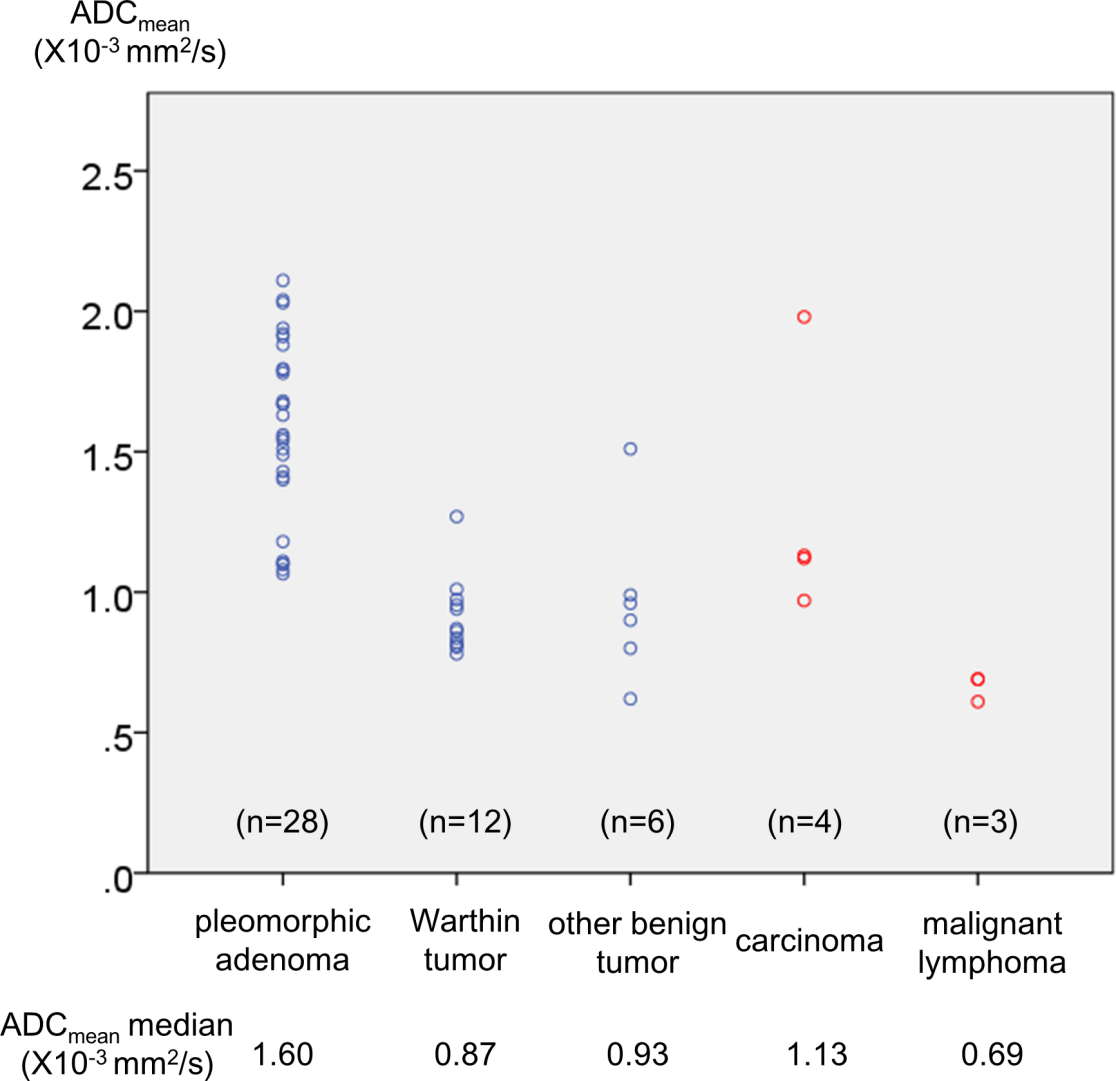
Scatter plots of ADC_mean_ for each histopathological category. The ADC ranges for pleomorphic adenoma, Warthin tumor, other benign tumors, carcinoma, and malignant lymphoma were 1.07 to 2.11 (median: 1.60) × 10^−3^, 0.78 to 1.27 (0.87) × 10^−3^, 0.62 to 1.51 (0.93) × 10^−3^, 0.97 to 1.98 (1.13) × 10^−3^, and 0.61 to 0.69 (0.69) × 10^−3^ mm^2^/sec, respectively.

Fifty-three patients (male:female = 28:25, age 21 to 84 years old, median: 54 years old) were enrolled in the study. The patient characteristics are shown in Table 1. Preoperative data and histopathological diagnosis and the final histopathological diagnosis are shown in Table 2. ADC_mean_ ranged from 0.61 to 2.11 (median: 1.13) × 10^−3^ mm^2^/sec. A scatter plots of ADC_mean_ for each histopathology is shown in Fig 2.

**Table 1:** Patient Characteristics.

Characteristic		No. of patients (%)
**Sex**		
	male	28 (53)
	female	25 (47)
**Histopathology**		
	pleomorphic adenoma	28 (53)
	Warthin tumor	12 (23)
	DLBCL	2(4)
	oncocytoma	2(4)
	inflammatory granulation	2(4)
	neurinoma	1(2)
	toxoplasma lymphadenitis	1(2)
	MALToma	1(2)
	SqCC	1(2)
	basal cell carcinoma	1(2)
	acinic cell carcinoma	1(2)
	salivary ductal carcinoma	1(2)
**Preoperative diagnostic group**		
	Group 1	20 (38)
	Group 2	12 (23)
	Group 3	21(39)

DLBCL: diffuse large B-cell lymphoma

MALToma: mucosa-associated lymphoid tissue lymphoma

SqCC: squamous cell carcinoma

**Table 2:** Preoperative examination results and histopathological diagnosis, and final histopathological diagnosis for each patient.

Patients					Pre-OP findings			Post-OP findings	Accuracy of pre-OP diagnosis	
Case	ADC_mean_	ROI area (mm^2^)	Age	Sex	Group	Additional Exam	Pre-OP Dx	Final histological Dx	Differential Dx of BT from MT	Histological Dx
1	2.04	225.4	46	M	1		PA	PA	○	○
2	0.97	116.7	57	M	2	Tc	WT	SDC	×	×
3	1.88	619.0	38	F	1		PA	PA	○	○
4	1.07	23.7	54	M	3	Tc+CNB	PA	PA	○	○
5	2.11	193.8	35	F	1		PA	PA	○	○
6	1.51	296.6	51	M	1		PA	PA	○	○
7	1.56	116.2	50	F	1		PA	PA	○	○
8	0.86	151.4	70	M	2	Tc	WT	WT	○	○
9	0.90	74.2	73	F	3	CNB	BT but PA	Onco	○	×
10	0.78	262.5	55	M	2	Tc	WT	WT	○	○
11	0.62	93.9	45	F	3	CNB	Epi Gra by Toxo	Toxo LA	○	○
12	2.03	151.3	60	M	1		PA	PA	○	○
13	1.13	199.7	69	M	3	Tc+CNB	ACC or AC	BCA	○	×
14	0.94	297.6	60	M	2	Tc	WT	WT	○	○
15	1.41	137.9	29	M	3	CNB	PA	PA	○	○
16	0.87	387.6	52	M	2	Tc	WT	WT	○	○
17	1.18	49.6	42	F	3	CNB	PA	PA	○	○
18	0.80	184.4	63	M	2	Tc	WT	WT	○	○
19	1.12	627.4	80	M	3	Tc+CNB	SqCC	SqCC	○	○
20	1.79	116.2	63	F	1		PA	PA	○	○
21	0.95	199.7	69	F	3	CNB	WT	WT	○	○
22	0.97	162.2	72	M	2	Tc	WT	WT	○	○
23	0.84	209.0	49	M	2	Tc	WT	WT	○	○
24	0.80	8.5	82	M	3	Tc+CNB	Gra-inf	Gra-inf	○	○
25	1.11	74.2	61	F	3	CNB	PA	PA	○	○
26	1.67	351.5	84	F	1		PA	PA	○	○
27	0.99	155.7	70	M	3	Tc+CNB	Gra-inf	Gra-inf	○	○
28	1.51	363.9	64	M	1		PA	Sch	○	×
29	1.80	310.0	49	M	1		PA	PA	○	○
30	1.27	541.4	51	M	2	Tc	WT	WT	○	○
31	1.98	43.0	47	F	1		PA	AcCC	×	×
32	1.55	458.8	44	M	1		PA	PA	○	○
33	0.81	161.7	70	M	2	Tc	WT	WT	○	○
34	1.54	68.7	50	F	1		PA	PA	○	○
35	1.68	193.3	50	F	1		PA	PA	○	○
36	0.61	376.7	45	F	3	CNB	ML	ML	○	○
37	1.67	602.2	54	M	1		PA	PA	○	○
38	1.94	225.4	75	F	1		PA	PA	○	○
39	1.08	92.4	43	F	3	CNB	PA	PA	○	○
40	0.69	327.3	82	F	3	CNB	ML	ML	○	○
41	1.91	82.6	53	F	1		PA	PA	○	○
42	1.63	84.5	38	M	1		PA	PA	○	○
43	1.01	88.0	71	M	2	Tc	WT	WT	○	○
44	1.10	177.0	61	M	3	Tc+CNB	PA	PA	○	○
45	0.82	91.6	40	M	2	Tc	WT	WT	○	○
46	1.92	141.4	72	F	1		PA	PA	○	○
47	1.49	575.5	21	F	3	CNB	PA	PA	○	○
48	1.40	391.1	61	M	3	Tc+CNB	PA	PA	○	○
49	0.69	111.2	80	F	3	CNB	ML	ML	○	○
50	1.10	161.2	48	F	3	CNB	PA	PA	○	○
51	1.78	101.3	46	F	1		PA	PA	○	○
52	0.96	102.0	66	F	3	CNB	Onco or WT	Onco	○	○
53	1.43	196.3	31	F	3	CNB	PA	PA	○	○

ADC: apparent diffusion coefficient, ROI: region of interest, Exam: examination, OP: operation, Dx: diagnosis, BT: benign tumor, MT: malignant tumor, CNB: core needle biopsy, Tc: 99mTc pertechnetate scintigraphy, PA: pleomorphic adenoma, WT: Warthin tumor, BT: benign tumor, Epi: epithelioid, Gra: granuloma, Toxo: toxoplasma, ACC: adenoid cystic carcinoma, AC: adenocarcinoma, SqCC: squamouc cell carcinoma, Gra-inf: granulomatous inflammation, ML: malignant lymphoma, Onco: oncocytoma, SDC: salivary ductal carcinoma, LA: lymphadenitis, BCA: basal cell adenocarcinoma, Sch: schwannoma, AcCC: acinici cell carcinoma

Nineteen patients underwent ^99m^Tc pertechnetate scintigraphy. Planar images were acquired for all 19 patients and SPECT/CT images were acquired for all but one patient (case 2), who underwent SPECT. Twelve of the 19 cases showed positive findings. Twenty-one of the 53 patients (40%) underwent CNB. No cases of tumor seeding, facial nerve palsy, infection, or hematoma occurred after CNB. In 7 cases, insufficient tissue was collected in CNB, and diagnosis was subsequently based on cytology (cases 9, 13, 24, 36, 39, 40 and 44).

Final histopathology after surgery showed that 28 cases (53%) were PA; 12 (23%) were WT; and 13 (24%) were other tumors, including 7 malignant tumors.

### Validation of the algorithm

**Fig 3:**
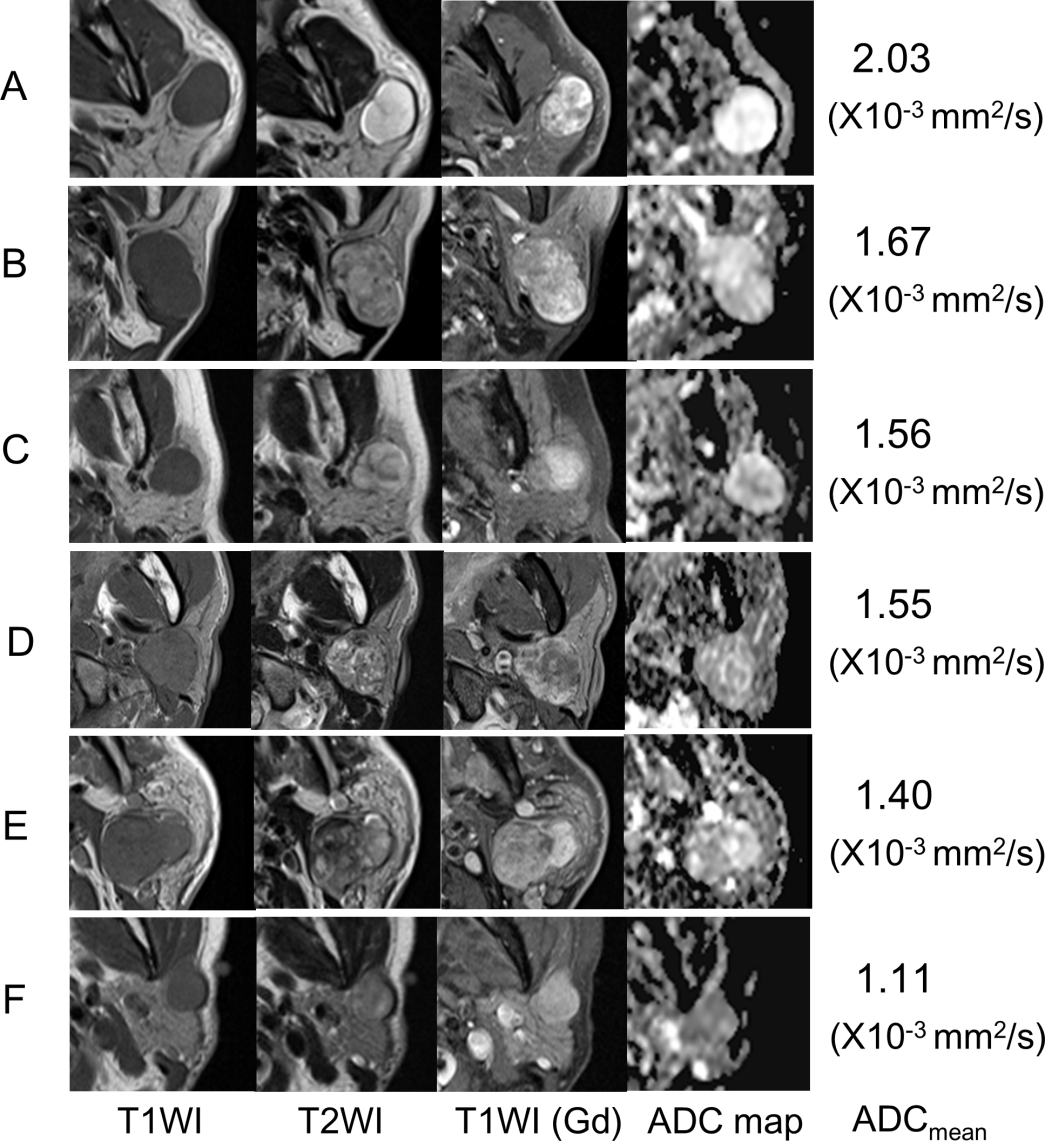
MRI of representative cases with pleomorphic adenoma. Tumors on the right side are reversed to the left side for easier viewing). Case A showed typical MRI findings of pleomorphic adenoma: homogenous T1 low-intensity, homogenous T2 hyperintensity, well-circumscribed borders, solid contrast enhancement, and a high signal on the ADC map. In contrast, cases B to E had indeterminate imaging features of heterogenous intermediate to low-intensity on T2WI, which overlap with malignant lesions. Note that even such indeterminate cases had a relatively high signal on the ADC map, suggestive of pleomorphic adenoma. Case F showed atypical images of pleomorphic adenoma with homogenous T1 low-intensity, heterogenous T2 intermediate to low-intensity, and a low signal on the ADC map, suggestive of non-pleomorphic adenoma. Cases A to D were classified into group 1, whereas cases E and F were classified into group 3. A: Case 12 (60-year-old man), B: Case 26 (84-year-old woman), C: Case 7 (50-year-old woman), D: Case 32 (44-year-old man), E: Case 48 (61-year-old man), F: Case 25 (61-year-old woman)

Diagnostic accuracy for PA: There were 28 PA cases at the final diagnosis. The ADC_mean_ of these cases ranged from 1.07 to 2.11 mm^2^/sec, with a median of 1.60 × 10^−3^ mm^2^/sec, and 18 had an ADC_mean_ ≥1.5 × 10^−3^ mm^2^/sec and were categorized in group 1. Based on a true-positive being a patient in group 1 with a final diagnosis of PA, the sensitivity, specificity, PPV, and NPV were 64%, 92%, 90%, and 70% respectively (Table 3). Representative MRI findings for PA cases are shown in Fig 3.

**Table 3:** Accuracy of preoperative diagnosis based on the algorithm.

	Group 1 s/o PA	Group 2 s/o WT	Group 3 s/o non-PA, non-WT	Total
**Post-OP histopathologic Dx**	(n)	(n)	(n)	(n)
PA	18	0	10	28
WT	0	11	1	12
non-PA, non-WT	2	1	10	13
**Diagnostic accuracy of Group 1 / 2**				
Sensitivity	64	92	―	―
Specificity	92	95	―	―
Positive predictive value	90	92	―	―
Negative predictive value	70	95	―	―
**CNB accuracy (Group 3)**			(%)	
Differential Dx between benign and malignant				
benign	―	―	100	―
malignant	―	―	100	―
total	―	―	100	―
Differential Dx in histopathology				
benign	―	―	81	―
malignant	―	―	80	―
total	―	―	81	―
**Accuracy of pre-OP Dx based on the algorithm**	(%)	(%)	(%)	(%)
Differential Dx between benign and malignant	95	92	100	96
Differential Dx in histopathology	90	92	81	87

PA: pleomorphic adenoma, WT: Warthin tumor, OP: operative, Dx: diagnosis, CNB: core needle biopsy

**Fig 4:**
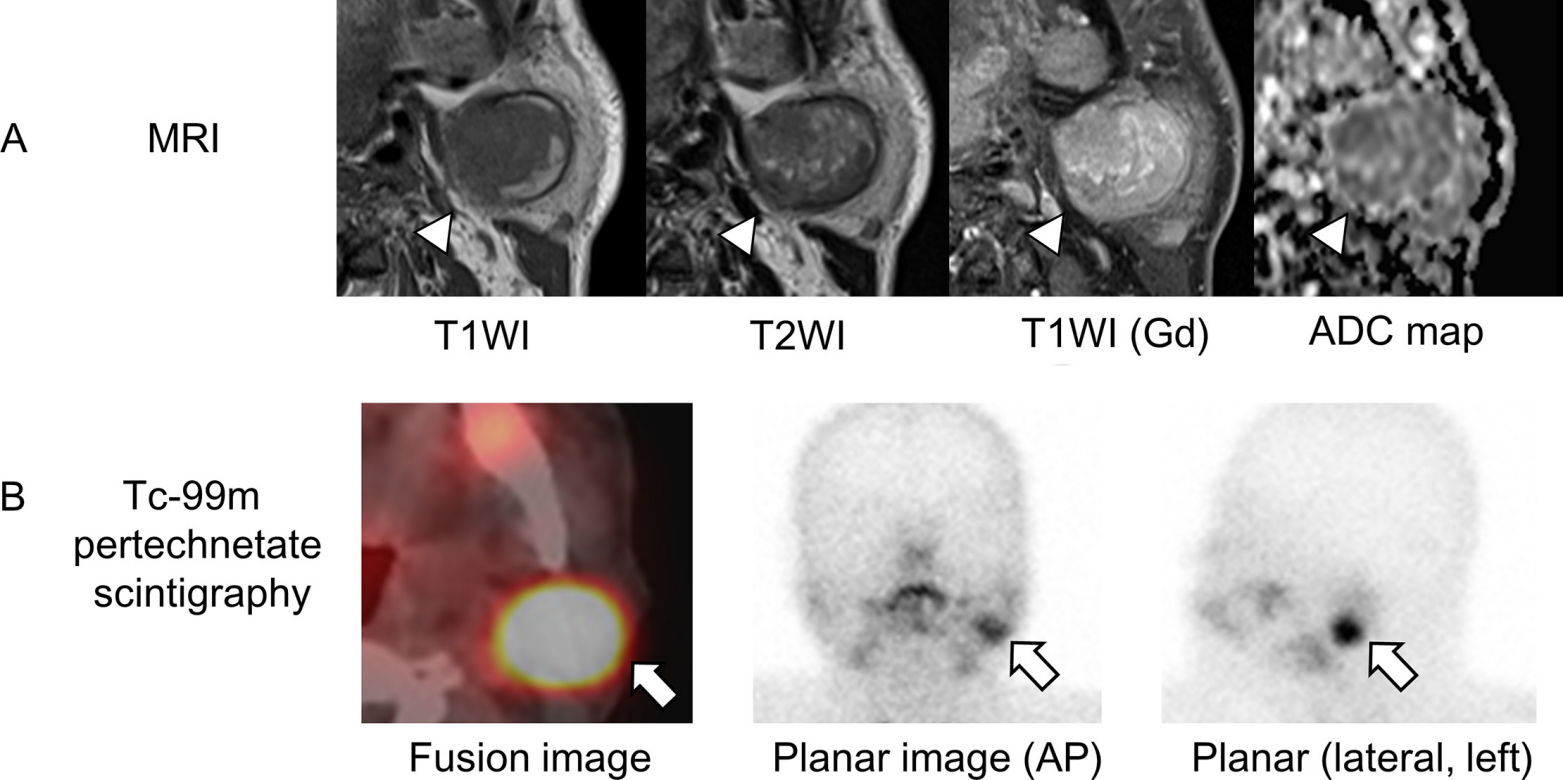
MRI and ^99m^Tc pertechnetate scintigraphy in a representative case of Warthin tumor. Warthin tumor on the left side of a 60-year-old man (case 14). (A) Axial MRI (white arrowheads) showed heterogenous (low to high) intensity on T1WI, heterogenous (low to intermediate) intensity on T2WI, strong gadolinium enhancement, and a low signal on the ADC map (ADC_mean_ 0.94 × 10^−3^ mm^2^/sec). (B) A fusion image (bottom left) and planar scans (bottom middle and right) in ^99m^Tc pertechnetate scintigraphy showed intense uptake (white arrows) in the tumor.

Diagnostic accuracy for WT: Twelve patients had a final diagnosis of WT, including 11 males over 40 years old and one female of age 69 years old (case 21). Only the female patient did not undergo ^99m^Tc pertechnetate scintigraphy, which led to a false-negative result. Among 33 patients with ADC_mean_ <1.5 × 10^−3^ mm^2^/sec, 19 were males ≥40 years old who underwent ^99m^Tc pertechnetate scintigraphy. Of these 19 patients, 12 had positive results (including one false-positive) and were categorized in group 2. Based on a true-positive being a patient in group 2 with a final diagnosis of WT, the sensitivity, specificity, PPV, and NPV were 92%, 95%, 92% and 95%, respectively (Table 3). Representative MRI and ^99m^Tc pertechnetate scintigraphy images for WT are shown in Fig 4.

**Fig 5:**
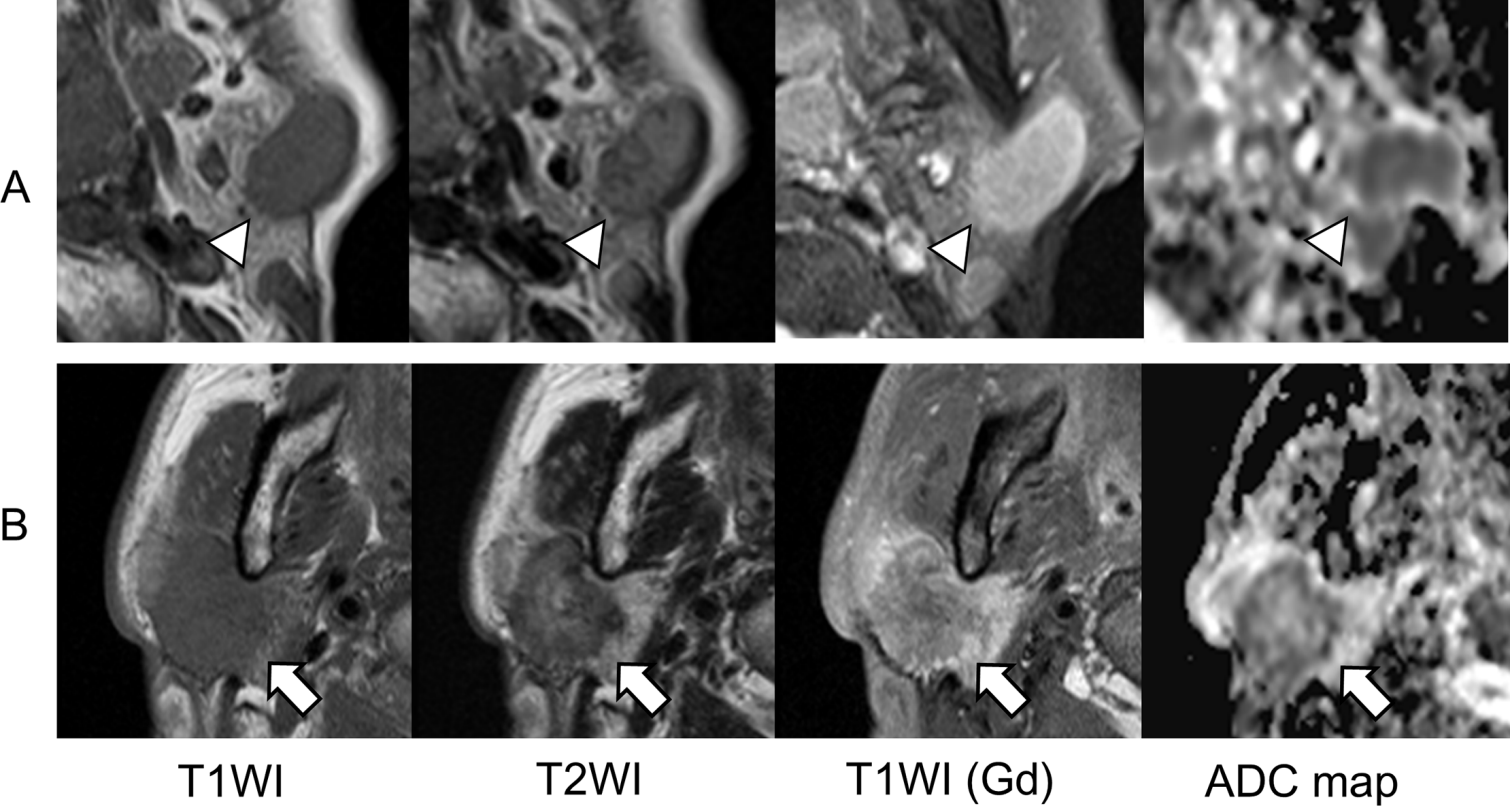
MRI findings in representative cases with malignant tumor. (A) Images of a diffuse large B cell lymphoma in the left parotid gland in a 48-year-old man (case 49). Axial MRI (white arrowheads) showed low intensity on T1WI, isointensity on T2WI, strong gadolinium enhancement, and a very low signal on the ADC map (ADC_mean_ 0.69 × 10^−3^ mm^2^/sec). (B) Images of basal cell carcinoma in the right parotid gland in a 69-year-old man (case 13). Axial MRI (white arrows) showed low intensity on T1WI, heterogenous (low to moderately high) intensity on T2WI, heterogenous gadolinium enhancement with poorly defined margins, and a low signal on the ADC map (ADC_mean_ 1.13 × 10^−3^ mm^2^/sec).

Diagnostic accuracy of CNB: Of the 33 patients with ADC_mean_ <1.5×10^−3^ mm^2^/sec, 21 (7 with negative results on ^99m^Tc pertechnetate scintigraphy and 14 who did not undergo ^99m^Tc pertechnetate scintigraphy) were categorized in group 3 and received CNB. Thus, 40% (21/53) of all patients underwent CNB. Of these 21 cases, 10 (48%) were PA and 1 (4%) was WT, all of which were diagnosed correctly by CNB, and 10 (48%) were non-PA and non-WT. CNB showed 100% accuracy for determining whether a tumor was benign or malignant, while the diagnostic accuracy of histopathology was 81% (Table 3). Representative MRI findings are shown in Fig 5.

Accuracy of preoperative diagnosis based on the algorithm: Overall, we made a preoperative histopathological diagnosis with an accuracy of 87% (46/53) and we correctly diagnosed whether a tumor was benign or malignant with an accuracy of 96% (51/53) (Table 3). Preoperative surgical planning had to be changed during surgery (enucleation to total parotidectomy) in only one case (2%) (case 2).

## Discussion

In the algorithm used in this study, PA and non-PA cases were first distinguished using ADC_mean_ measured on diffusion-weighed MRI. Next, among suspected non-PA cases, WT and non-WT cases were distinguished by evaluation of epidemiology and subsequent ^99m^Tc pertechnetate scintigraphy. Finally, to make a histopathological diagnosis, CNB was performed with an 18-gauge needle only in patients who were found to be likely to be non-PA and non-WT cases in screening. This approach resulted in performance of CNB in only 40% of all patients, while the accuracy of the preoperative histopathological diagnosis was 87%. These results indicate that our algorithm for preoperative histopathological diagnosis and surgical planning for parotid gland tumors may be clinically useful with less risk of CNB procedure than a plan of just doing CNB on all parotid gland tumors.

Conventional MRI without DWI is useful for preoperative diagnosis of PA, with a high specificity of 95%[21] and PPV of 86–96%[21, 22]. If MRI findings for parotid gland tumors show homogenous T2 hyperintensity, well-circumscribed borders and solid contrast enhancement[22], diagnosis of PA is straightforward. However, a significant proportion of PA cases have indeterminate imaging features (heterogenous intermediate to low-intensity on T2WI), which overlap with those for malignant lesions, and this explains the low sensitivity of 43.9%[21].

Quantification of ADC using mapping is a useful diagnostic imaging method for differentiation of PA from non-PA because it allows diagnosis of a tumor with intermediate to low-intensity on T2WI, which may suggest a malignant tumor, as PA if the tumor has a high ADC_mean_[13–15, 23]. The average reported ADC_mean_ for PA ranges from 1.74 to 2.15×10^−3^ mm^2^/s[12–15], and our median value of 1.60×10^−3^ mm^2^/s is close to this range. DWI may also be useful for distinguishing PA and non-PA cases with an ADC_mean_ cut-off of 1.315 to 1.4×10^−3^ mm^2^/s[12, 14], which is slightly lower than our cut-off of 1.5×10^−3^ mm^2^/s. In this study, we performed surgery without preoperative biopsy in patients with high ADC_mean_ (≥1.5×10^−3^ mm^2^/s) who were classified in group 1 based on suspected PA. Thus, we had to set the threshold at a higher level than that in previous reports[12, 14] to avoid classifying patients with malignant tumors into group 1. Thus, the sensitivity for diagnosis of PA using ADC_mean_ was relatively low (64%), but the specificity was high (92%). The false-negative rate (36%) was also high, but this was acceptable because all false-negative cases were diagnosed correctly by subsequent CNB. Quantitative estimation of ADC is practical clinically and is not as time-consuming as dynamic contrast-enhanced MRI, which is also useful for improved histological diagnosis of a parotid gland tumor[14, 24].

WT has a low ADC_mean_ of 0.72 to 0.96×10^-3^mm^2^/s[13, 14, 17, 18] due to its high cellularity caused by epithelial proliferation with lymphocytic infiltration[17]. Therefore, WT cannot be differentiated from malignant tumors using ADC_mean_. Thus, quantitative analysis of parotid gland tumors using ADC_mean_ permits differentiation of PA from non-PA, but cannot differentiate benign from malignant tumors. Epidemiologically, WT is more common in males over 40 years old[25] with a male-to-female ratio of 5 to 6.5:1[20, 25, 26]. ^99m^Tc pertechnetate scintigraphy is useful for diagnosis of WT, with Miyake al. finding a sensitivity for detection of WT on ^99m^Tc pertechnetate scintigrapy after lemon juice stimulation of 94%, a specificity of 94%, and an accuracy of 94%[19]. ^99m^Tc pertechnetate scintigraphy is not specific for WT, and false-positive results may occur due to partial obstruction of the parotid gland, while false-negative results may be due to large cystic changes[27]. However, even if WTs have relatively large cystic components, the sensitivity of ^99m^Tc pertechnetate scintigraphy becomes nearly 100% using tracer uptake patterns, such as non-homogenous (alternating hot and warm areas) and mixed (hot and cold areas), as positive results[20]. Therefore, it should be possible to diagnose WT among tumors with low ADC_mean_ (tumors diagnosed as non-PA by ADC_mean_ analysis) by evaluating the epidemiology (males ≥40 years old) and performing ^99m^Tc pertechnetate scintigraphy.

The sensitivity and specificity for diagnosis of WT based on the ADC_mean_, sex and age, and ^99m^Tc pertechnetate scintigraphy (if done) were 92% and 95%, respectively in this study. There was only one false-negative case: a female who did not undergo ^99m^Tc pertechnetate scintigraphy, but was diagnosed correctly by subsequent CNB. Therefore, we reduced the number of patients who underwent ^99m^Tc pertechnetate scintigraphy by evaluating ADC_mean_, sex and age. Indeed, for diagnosis of WT, we were able to increase the pretest probability from 23% (12/53) to 63% (12/19). This suggests that our algorithm for diagnosis of WT is useful, cost-effective and helpful for avoiding unnecessary radiation exposure.

Using the algorithm, we were able to identify patients with tumors that were likely to be non-PA and non-WT, and in whom CNB was required, thereby avoiding unnecessary CNB for patients with PA or WT. Of the 21 patients who underwent CNB (group 3), 10 (48%) were PA cases and 1 (5%) was WT, all of which were diagnosed correctly by CNB. Ideally, cases in group 3 should all have been non-PA and non-WT tumors, but only 48% (10/21) of cases in group 3 were non-PA and non-WT. Using a threshold of 1.4×10^−3^ mm^2^/s to differentiate PA from non-PA, 4 PA cases in group 3 would have been categorized in group 1, and the rate of non-PA and non-WT cases in group 3 would have increased from 48% to 59% (10/17). Thus, the number of patients who needed CNB would have been reduced from 40% (21/53) to 32% (17/53), and diagnosis of PA by ADC_mean_ would have been improved (sensitivity, specificity, PPV, and NPV of 71%, 92%, 91% and 74% respectively) using a threshold of 1.4×10^−3^ mm^2^/s, which suggests that this value may be more appropriate than 1.5×10^−3^ mm^2^/s.

CNB for the 21 patients in group 3 had 100% accuracy in determining whether a tumor was benign or malignant, but the diagnostic accuracy using histopathology was only 81%, which is lower compared to previous studies[3, 6, 7]. We did not perform CNB using a commercial needle device with a special cutting edge, but with a regular hollow 18-gauge needle. Thus, we may not have obtained as much tissue as that in CNB using a special cutting edge, and this may account for our slightly poorer outcomes in histopathological diagnosis. Although no cases of tumor seeding, facial nerve palsy, infection, or hematoma occurred in our study, there is a need to refine our CNB technique to improve the accuracy of preoperative diagnosis.

Of the 20 patients in group 1 diagnosed with PA before surgery, 2 were diagnosed with another type of tumor after surgery: schwannoma (case 28, Table 2) and acinic cell carcinoma (case 31, Table 2). Case 28 underwent extracapsular removal of the tumor, which was located in the deep portion of the parotid gland, and had an uneventful postoperative course. Case 31 underwent partial superficial parotidectomy and had a good postoperative course for 2 years. Chuang et al.[28] obtained a low ADC_mean_ for solid vestibular schwannomas, with an average of 1.06±0.17×10^-3^mm^2^/s. The case of schwannoma (case 28) in the current study had an ADC_mean_ of 1.51×10^-3^mm^2^/s, which might have been increased by micro-cysts in the tumor. Regardless, facial nerve schwannoma in the parotid gland is a relatively rare disease that does not have specific findings on MRI, and hence it is not practical to screen for such a rare benign tumor by DWI. Habermann et al. found a low mean ADC_mean_ of acinic cell carcinoma (n = 10), with an average of 0.79±0.33×10^−3^ mm^2^/s. In microscopic findings, micro-cysts, hemorrhage and necrosis are common in acinic cell carcinomas[29], and hence it is likely that these tumors with significant cystic or necrotic components will have a high ADC_mean_. We tried to place a ROI within solid portions of tumors (excluding clear necrotic or cystic regions with no enhancement on contrast-enhanced MRI) to obtain an appropriate estimation of ADC_mean_. However, in analysis of MRI of the acinic cell carcinoma (case 31) retrospectively, the tumor was not well enhanced, and had many micro-cysts in microscopic findings. Hence, this case should have been excluded from the study and we believe that it is unlikely that such a malignant tumor will normally fall into group 1 in our algorithm.

Of the 12 patients in group 2 (diagnosed with WT before surgery), one was diagnosed with salivary ductal carcinoma by intraoperative frozen section. This case (case 2) had a T1-low, T2-low to intermediate, and heterogeneously enhanced tumor in the deep portion of the gland, and the ADC_mean_ was 0.97×10^-3^mm^2^/s. The tumor displayed a mixed scan pattern (hot and cold areas)[20] in planar ^99m^Tc pertechnetate scintigraphy, which led to diagnosis of WT. Retrospectively, this false-positive scan seemed to result from partial obstruction of the parotid gland. This was the only case diagnosed using planar images; all subsequent cases were diagnosed by SPECT/CT. In general, SPECT/CT images allow more accurate identification of lesions and thus we propose to use this method preoperatively to diagnose WT. Moreover, CNB should be performed in a case with equivocal findings on ^99m^Tc scintigraphy.

The current study has several limitations. First, regarding the exclusion criteria, we excluded patients with pure cyst or tumor with a necrotic/cystic component >50% of the total lesion volume (n = 4) and patients with pain or facial paralysis that were strongly suggestive of malignancy (n = 1). It may have led to patient selection bias. However, we previously reported that most of the parotid gland tumors which show cystic change are benign WT[30]. As we mentioned before, even if WTs have relatively large cystic components, the sensitivity of ^99m^Tc pertechnetate scintigraphy would become nearly 100% regarding tracer uptake patterns such as non-homogenous and mixed as positive results. This means that excluding tumors with a necrotic/cystic component>50% would not have influenced the diagnostic performance of the scintigraphy for WTs in the study. In addition, among malignant parotid gland tumors, most of the tumors which show cystic degeneration are low grade carnicoma such as acinic cell carcinoma and low grade mucoepidermoid carcinoma[30]. This indicates that patients who have tumors with predominant cystic formation may not need the CNB procedure because those tumors are not likely to be high grade carcinoma, which should be thoroughly diagnosed before surgery using CNB. In cases who represent pain or facial paralysis that are strongly suggestive of malignancy, we suppose they should be undergone CNB as soon as possible in order not to be late for therapy.

Second, the population of malignancy is rather small in this study (13%, 7/53). This might restrict the statistical power of the sensitivity for diagnosing malignant lesions. In fact, two malignant tumors were misclassified as benign tumors, making the sensitivity values for malignant lesion were only 71.4%. In addition, our algorithm seems to be extreme because 60% of patients did not undergo any biopsy including FNAC before surgery. If we would have performed FNAC on all parotid tumors, instead of our algorithm, the two tumors might have been truly classified as malignant tumors. We don’t think it’s necessary to perform FNAC on all parotid gland tumors because most of PA can be diagnosed by ADC analysis and most of WT can be diagnosed by ADC analysis and ^99m^Tc pertechnetate scintigraphy. In order not to misclassify malignant tumor as benign tumor, however, we may perform FNAC or CNB additionally for patients in group 1 or 2 with indeterminate findings of MRI (i.e. when ADC is close to the cut-off value or when it is difficult to differentiate tumor from cystic lesion) or ^99m^Tc pertechnetate scintigraphy (i.e. when the degree of uptake is equivocal).

Third, the cut-off value of 1.5×10^-3^mm^2^/s for PA and non-PA in our algorithm may not have been evidence based. The previous papers reported that an ADC_mean_ cut-off of 1.315 to 1.4×10^−3^ mm^2^/s is useful for distinguishing PA and non-PA[12, 14]. We set the cut-off value at slightly higher level to decrease the possibility of classifying patients with malignant tumors into group 1, however, the choice of preset value might be arbitrary. It would have been better if set at 1.4×10^-3^mm^2^/s, based on the previous report[14, 31], as that appeared in the discussion.

Fourth, we performed ^99m^Tc pertechnetate scintigraphy only on patients who were males ≥40 years old for distinguishing WT and non-WT. This is because 93% of WT patients are ≥40 years old and a male-to-female ratio is high (male:female = 5.5~6.5:1) in Japan[25, 26]. However, increasing incidence for females has also been reported (male:female = 2:1) in other countries[31, 32], which suggests that the prevalence and epidemiology of WT may be variable among different races. Therefore, our algorithm of distinguishing WT and non-WT may not be in clinical use in other countries.

Finally, it was difficult in some cases to obtain perfect image registration between the different image sequences. We performed the DWI exams with conventional EPI sequence, which sometimes causes geometric distortion. In the future study, a recently developed technique, readout segmented EPI could help to reduce the distortion and to improve the image quality[33, 34].

However, the study is of value as what we believe to be the first prospective examination of a preoperative diagnostic strategy using diffusion-weighed MRI with calculation of ADC_mean_ and ^99m^Tc pertechnetate scintigraphy in parotid gland tumors. Use of an algorithm for preoperative screening of patients with parotid gland tumors that were likely to be non-PA and non-WT permitted CNB to be performed in only 40% of patients, while giving a preoperative histopathological diagnosis with an accuracy of 87%. These results suggest that our algorithm is reliable, efficient and possessing less risk of CNB procedure than a plan of just doing CNB on all parotid gland tumors. Its clinical utility requires further evaluation in larger studies.

## References

[1] Seethala RR, LiVolsi VA, Baloch ZW. Relative accuracy of fine-needle aspiration and frozen section in the diagnosis of lesions of the parotid gland. Head & neck. 2005;27(3):217–23. 10.1002/hed.20142 . 15672359

[2] Zbaren P, Nuyens M, Loosli H, Stauffer E. Diagnostic accuracy of fine-needle aspiration cytology and frozen section in primary parotid carcinoma. Cancer. 2004;100(9):1876–83. 10.1002/cncr.20186 . 15112268

[3] Witt BL, Schmidt RL. Ultrasound-guided core needle biopsy of salivary gland lesions: a systematic review and meta-analysis. The Laryngoscope. 2014;124(3):695–700. 10.1002/lary.24339 . 23929672

[4] Cho HW, Kim J, Choi J, Choi HS, Kim ES, Kim SH, et al Sonographically guided fine-needle aspiration biopsy of major salivary gland masses: a review of 245 cases. AJR American journal of roentgenology. 2011;196(5):1160–3. Epub 2011/04/23. 10.2214/ajr.10.4256 . 21512086

[5] Schmidt RL, Hall BJ, Wilson AR, Layfield LJ. A systematic review and meta-analysis of the diagnostic accuracy of fine-needle aspiration cytology for parotid gland lesions. American journal of clinical pathology. 2011;136(1):45–59. Epub 2011/06/21. 10.1309/ajcpoie0cznat6sq . 21685031

[6] Haldar S, Mandalia U, Skelton E, Chow V, Turner SS, Ramesar K, et al Diagnostic investigation of parotid neoplasms: a 16-year experience of freehand fine needle aspiration cytology and ultrasound-guided core needle biopsy. International journal of oral and maxillofacial surgery. 2014 10.1016/j.ijom.2014.09.025 . 25457828

[7] Douville NJ, Bradford CR. Comparison of ultrasound-guided core biopsy versus fine-needle aspiration biopsy in the evaluation of salivary gland lesions. Head & neck. 2013;35(11):1657–61. 10.1002/hed.23193 . 23109044

[8] Wu EH, Chen YL, Wu YM, Huang YT, Wong HF, Ng SH. CT-guided core needle biopsy of deep suprahyoid head and neck lesions. Korean journal of radiology: official journal of the Korean Radiological Society. 2013;14(2):299–306. Epub 2013/03/14. 10.3348/kjr.2013.14.2.299 ; PubMed Central PMCID: PMCPmc3590344. 23482651 PMC3590344

[9] Pfeiffer J, Ridder GJ. Diagnostic value of ultrasound-guided core needle biopsy in patients with salivary gland masses. International journal of oral and maxillofacial surgery. 2012;41(4):437–43. Epub 2011/12/30. 10.1016/j.ijom.2011.12.005 . 22204925

[10] Srinivasan A, Dvorak R, Perni K, Rohrer S, Mukherji SK. Differentiation of benign and malignant pathology in the head and neck using 3T apparent diffusion coefficient values: early experience. AJNR American journal of neuroradiology. 2008;29(1):40–4. 10.3174/ajnr.A0743 . 17921228 PMC8119114

[11] Sasaki M, Eida S, Sumi M, Nakamura T. Apparent diffusion coefficient mapping for sinonasal diseases: differentiation of benign and malignant lesions. AJNR American journal of neuroradiology. 2011;32(6):1100–6. Epub 2011/03/12. 10.3174/ajnr.A2434 . 21393402 PMC8013129

[12] Celebi I, Mahmutoglu AS, Ucgul A, Ulusay SM, Basak T, Basak M. Quantitative diffusion-weighted magnetic resonance imaging in the evaluation of parotid gland masses: a study with histopathological correlation. Clinical imaging. 2013;37(2):232–8. Epub 2013/03/08. 10.1016/j.clinimag.2012.04.025 . 23465973

[13] Habermann CR, Arndt C, Graessner J, Diestel L, Petersen KU, Reitmeier F, et al Diffusion-weighted echo-planar MR imaging of primary parotid gland tumors: is a prediction of different histologic subtypes possible? AJNR American journal of neuroradiology. 2009;30(3):591–6. Epub 2009/01/10. 10.3174/ajnr.A1412 . 19131405 PMC7051445

[14] Yabuuchi H, Matsuo Y, Kamitani T, Setoguchi T, Okafuji T, Soeda H, et al Parotid gland tumors: can addition of diffusion-weighted MR imaging to dynamic contrast-enhanced MR imaging improve diagnostic accuracy in characterization? Radiology. 2008;249(3):909–16. Epub 2008/10/23. 10.1148/radiol.2493072045 . 18941162

[15] Yerli H, Agildere AM, Aydin E, Geyik E, Haberal N, Kaskati T, et al Value of apparent diffusion coefficient calculation in the differential diagnosis of parotid gland tumors. Acta Radiol. 2007;48(9):980–7. Epub 2007/10/25. 10.1080/02841850701501717 . 17957512

[16] Maeda M, Maier SE. Usefulness of diffusion-weighted imaging and the apparent diffusion coefficient in the assessment of head and neck tumors. Journal of neuroradiology Journal de neuroradiologie. 2008;35(2):71–8. 10.1016/j.neurad.2008.01.080 . 18325591

[17] Ikeda M, Motoori K, Hanazawa T, Nagai Y, Yamamoto S, Ueda T, et al Warthin tumor of the parotid gland: diagnostic value of MR imaging with histopathologic correlation. AJNR American journal of neuroradiology. 2004;25(7):1256–62. Epub 2004/08/18. . 15313720 PMC7976549

[18] Yoshino N, Yamada I, Ohbayashi N, Honda E, Ida M, Kurabayashi T, et al Salivary glands and lesions: evaluation of apparent diffusion coefficients with split-echo diffusion-weighted MR imaging—initial results. Radiology. 2001;221(3):837–42. Epub 2001/11/24. 10.1148/radiol.2213010131 . 11719687

[19] Miyake H, Matsumoto A, Hori Y, Takeoka H, Kiyosue H, Hori Y, et al Warthin's tumor of parotid gland on Tc-99m pertechnetate scintigraphy with lemon juice stimulation: Tc-99m uptake, size, and pathologic correlation. European radiology. 2001;11(12):2472–8. Epub 2001/12/06. 10.1007/s003300100839 . 11734943

[20] Weinstein GS, Harvey RT, Zimmer W, Ter S, Alavi A. Technetium-99m pertechnetate salivary gland imaging: its role in the diagnosis of Warthin's tumor. Journal of nuclear medicine: official publication, Society of Nuclear Medicine. 1994;35(1):179–83. Epub 1994/01/01. . 8271043

[21] Zaghi S, Hendizadeh L, Hung T, Farahvar S, Abemayor E, Sepahdari AR. MRI criteria for the diagnosis of pleomorphic adenoma: a validation study. American journal of otolaryngology. 2014;35(6):713–8. Epub 2014/08/19. 10.1016/j.amjoto.2014.07.013 . 25128908

[22] Heaton CM, Chazen JL, van Zante A, Glastonbury CM, Kezirian EJ, Eisele DW. Pleomorphic adenoma of the major salivary glands: Diagnostic utility of FNAB and MRI. The Laryngoscope. 2013 10.1002/lary.24247 . 23798340

[23] Eida S, Sumi M, Sakihama N, Takahashi H, Nakamura T. Apparent diffusion coefficient mapping of salivary gland tumors: prediction of the benignancy and malignancy. AJNR American journal of neuroradiology. 2007;28(1):116–21. Epub 2007/01/11. . 17213436 PMC8134115

[24] Motoori K, Ueda T, Uchida Y, Chazono H, Suzuki H, Ito H. Identification of Warthin tumor: magnetic resonance imaging versus salivary scintigraphy with technetium-99m pertechnetate. Journal of computer assisted tomography. 2005;29(4):506–12. Epub 2005/07/14. . 16012309 10.1097/01.rct.0000164672.34261.33

[25] Ibi A, Yokobayashi T, Kawasaki T, Nakajima T. Bilateral Warthin's tumor: report of case and review of Japanese literature. Journal of oral surgery (American Dental Association: 1965). 1981;39(5):362–6. Epub 1981/05/01. . 6938654

[26] Ichihara T, Kawata R, Higashino M, Terada T, Haginomori S. A more appropriate clinical classification of benign parotid tumors: investigation of 425 cases. Acta oto-laryngologica. 2014;134(11):1185–91. Epub 2014/10/16. 10.3109/00016489.2014.914246 . 25315918

[27] Chapnik JS. The controversy of Warthin's tumor. The Laryngoscope. 1983;93(6):695–716. Epub 1983/06/01. . 6304434 10.1288/00005537-198306000-00002

[28] Chuang CC, Chang CS, Tyan YS, Chuang KS, Tu HT, Huang CF. Use of apparent diffusion coefficients in evaluating the response of vestibular schwannomas to Gamma Knife surgery. Journal of neurosurgery. 2012;117 Suppl:63–8. Epub 2012/12/12. 10.3171/2012.7.gks121003 . 23205791

[29] Suh SI, Seol HY, Kim TK, Lee NJ, Kim JH, Kim KA, et al Acinic cell carcinoma of the head and neck: radiologic-pathologic correlation. Journal of computer assisted tomography. 2005;29(1):121–6. Epub 2005/01/25. . 15665697 10.1097/01.rct.0000150141.14113.ab

[30] Kikuchi M, Shinohara S, Fujiwara K, Hori SY, Tona Y, Yamazaki H, et al [Cystic parotid gland lesion evaluation]. Nihon Jibiinkoka Gakkai kaiho. 2010;113(5):441–9. Epub 2010/06/22. . 20560405 10.3950/jibiinkoka.113.441

[31] Peter Klussmann J, Wittekindt C, Florian Preuss S, Al Attab A, Schroeder U, Guntinas-Lichius O. High risk for bilateral Warthin tumor in heavy smokers—review of 185 cases. Acta oto-laryngologica. 2006;126(11):1213–7. 10.1080/00016480600740605 . 17050316

[32] Vories AA, Ramirez SG. Warthin's tumor and cigarette smoking. Southern medical journal. 1997;90(4):416–8. Epub 1997/04/01. . 9114834 10.1097/00007611-199704000-00011

[33] Juan CJ, Chang HC, Hsueh CJ, Liu HS, Huang YC, Chung HW, et al Salivary glands: echo-planar versus PROPELLER Diffusion-weighted MR imaging for assessment of ADCs. Radiology. 2009;253(1):144–52. Epub 2009/10/01. 10.1148/radiol.2531082228 . 19789257

[34] Koyasu S, Iima M, Umeoka S, Morisawa N, Porter DA, Ito J, et al The clinical utility of reduced-distortion readout-segmented echo-planar imaging in the head and neck region: initial experience. European radiology. 2014;24(12):3088–96. Epub 2014/08/15. 10.1007/s00330-014-3369-5 . 25117744

